# Using global feedback to induce learning of gist of abnormality in mammograms

**DOI:** 10.1186/s41235-022-00457-8

**Published:** 2023-01-08

**Authors:** E. M. Raat, C. Kyle-Davidson, K. K. Evans

**Affiliations:** University of York, Heslington, York, YO10 5DD UK

**Keywords:** Gist of abnormality, Gist extraction, Medical image perception, Medical expertise, Medical imaging, Perceptual learning, Implicit learning, Statistical learning, Deep neural network

## Abstract

Extraction of global structural regularities provides general ‘gist’ of our everyday visual environment as it does the gist of abnormality for medical experts reviewing medical images. We investigated whether naïve observers could learn this gist of medical abnormality. Fifteen participants completed nine adaptive training sessions viewing four categories of unilateral mammograms: normal, obvious-abnormal, subtle-abnormal, and global signals of abnormality (mammograms with no visible lesions but from breasts contralateral to or years prior to the development of cancer) and receiving only categorical feedback. Performance was tested pre-training, post-training, and after a week’s retention on 200 mammograms viewed for 500 ms without feedback. Performance measured as d’ was modulated by mammogram category, with the highest performance for mammograms with visible lesions. Post-training, twelve observed showed increased d’ for all mammogram categories but a subset of nine, labelled learners also showed a positive correlation of d’ across training. Critically, learners learned to detect abnormality in mammograms with only the global signals, but improvements were poorly retained. A state-of-the-art breast cancer classifier detected mammograms with lesions but struggled to detect cancer in mammograms with the global signal of abnormality. The gist of abnormality can be learned through perceptual/incidental learning in mammograms both with and without visible lesions, subject to individual differences. Poor retention suggests perceptual tuning to gist needs maintenance, converging with findings that radiologists’ gist performance correlates with the number of cases reviewed per year, not years of experience. The human visual system can tune itself to complex global signals not easily captured by current deep neural networks.

Medical experts often report having a gut feeling about the state of a radiograph when briefly looking at certain medical imaging cases, where they get the impression that something might be wrong but are not able to pinpoint the exact image elements that made them feel that way. These anecdotes suggest medical experts might rapidly access first impressions of abnormality. However, there is more than just anecdotal evidence for this notion: it is also supported by human observer studies, which have shown that radiologists are able to discriminate between normal and abnormal medical images with above-chance accuracy within 200–500 ms for chest radiographs (Kundel & Nodine, 1975), pathology images, or mammograms (Evans et al., 2013a, 2013b), the latter of which will be the focus of the current study. Thus, medical experts indeed possess the perceptual ability to rapidly extract a signal that indicates abnormality from images in their field of expertise.

This shows an incredible perceptive power, which is furthered by research demonstrating that the ability does not rely on the presence of a localizable signal like a lesion. Indeed, radiologists can recognize this gist of abnormality in patches of the abnormal mammogram that do not contain a lesion, or even from the breast contralateral to the abnormality (Evans et al., 2016), both of which do not contain any localizable abnormalities. Even more striking, when normal mammograms from women who went on to develop cancer in the next two to three years were intermixed with normal and abnormal mammograms, they were rated as significantly more abnormal than the normal images (Brennan et al., 2018; Evans et al., 2019). Thus, the gist of abnormality signal can be detected without localizable abnormalities. For mammograms containing a single mass, it has been suggested that radiologists can sometimes access coarse location information (Carrigan et al., 2018), although this study did remove image artefacts and large calcifications from the breast tissue. Together, these findings point to a rapidly extracted global signal of image statistics that allows medical experts to detect whether the imaged tissue is normal or abnormal, which might provide access to coarse location information, but does not require local information to function. This description fits closely with the process of gist extraction that has been widely described in the scene processing literature.

Gist extraction is a perceptual process that allows observers to quickly retrieve the global meaning, or gist, of visual input. After as little as 20–30 ms, humans can accurately discriminate between man-made and natural environments, so-called superordinate categories (Joubert et al., 2009), recognize forests, fields, rivers, and other basic scene categories (Greene & Oliva, 2009), or determine the presence or absence of broad categories such as animals (Bacon-Macé et al., 2005) or vehicles (VanRullen & Thorpe, 2001). Indeed, there is a wide range of research showing that humans can extract surprisingly complex information from rapidly presented visual information, which fits closely with the observations in rapid medical image perception.

The key characteristics of gist extraction are that it occurs rapidly, globally (across the whole image) with a loss of specific local information and does not require focused attention. Instead, it occurs without prior location of items and in a non-selective manner. For example, gist can be extracted from scenes in the periphery in parallel with a demanding foveal letter discrimination task (Li et al., 2003) or from two, or even four scenes in parallel with minimal drops in performance (Rousselet et al., 2004) or scenes presented in medium-to-far periphery (Boucart et al., 2013; Larson & Loschky, 2009), clearly showcasing the global and non-selective nature of the process. In addition, gist extraction does not require prior configuration of the visual system: it occurs when monitoring for multiple cue categories simultaneously (Evans et al., 2011a, 2011b), or even when the target category is post-cued after a rapid serial visual presentation (Evans et al., 2011a, 2011b; Potter et al., 2014). However, it also means that information about the locations of specific elements that make up the scene is not consciously accessible (Evans & Treisman, 2005). Overall, scene gist extraction clearly occurs rapidly, globally, and without the need of focused attention or preselection, which fits closely with the observations of what we will refer to as the *gist of (medical) abnormality.*

But which signals are extracted by this global, rapid process to contribute to the formation of our gist understanding? As every image is built up from spatial frequencies at various orientations, shared categorical regularities between a gist category might be captured in similarities in spatial structural regularities, as described by Portilla and Simoncelli (2000)’s statistics. The statistic they define are extracted using spatial filters of specific sizes and orientations and are applied to noise to create an artificial ‘metamer’, that contains the same spatial structural regularities, but no recognizable objects. Such a metamer is indistinguishable from the original in two alternative forced choice tasks (2-AFC) at 200 ms viewing time (Freeman & Simoncelli, 2011), suggesting that spatial structural regularities capture essential aspects of scenes that are accessed during gist extraction. The idea of a statistical signature of an image fits with the Efficient Coding Hypothesis (Simoncelli, 2003), as reducing an image to its spatial structural regularities would allow efficient encoding of its essential information. Mammogram content is even more closely related to its spatial frequency content than scene images, due to most of the content being textural. For example, previous research has shown that low-pass filtering strongly reduced gist extraction, while high-pass filtered mammograms retained most gist information (Evans et al., 2016). Spatial structural regularities might be more similar between images from the same category and thus allow for flexible perceptual rules for gist categorization.

Oliva and Torralba (2001) further explained these spatial structural regularities with a focus on human perception through gist descriptors, which similarly captured spatial frequency patterns on a global spatial scale, the global spatial envelope. Gist descriptors can be represented as scores on scales such as expansiveness and openness. Patterns in these feature scores have been shown to be more similar within than between scene categories. Additionally, false alarms made by observers could often be predicted by similarities in gist predictors (Greene & Oliva, 2009). This supports the idea that shared patterns of frequencies and textures could play an important role in the flexible, yet reliable gist categorization of scenes, which could reasonably be extended to mammograms.

To allow for its non-selective and global nature, gist extraction must be highly flexible, especially as it must generalize across a wide range of exemplars that all fall under one gist category. For example, we can recognize the gist category of a scene environment in a variety of conditions, such as viewing angles, lighting, and specific objects (Fig. 1A, B), and the same applies to mammograms, as these can also vary widely in their appearance, size, shape, density, and texture (Fig. 1C, D). However, previous experience influences our ability to extract gist accurately, as human observers performed considerably worse on scene gist extraction for photographs from aerial compared to terrestrial viewpoints (Loschky et al., 2015). Thus, our brain might develop a set of general perceptual rules of expected spatial regularities for each gist category, based on previous experience, that are flexible enough to generalize across variations, but specific enough to allow it to distinguish a beach from a river, or a normal from an abnormal mammogram.

**Fig. 1:**
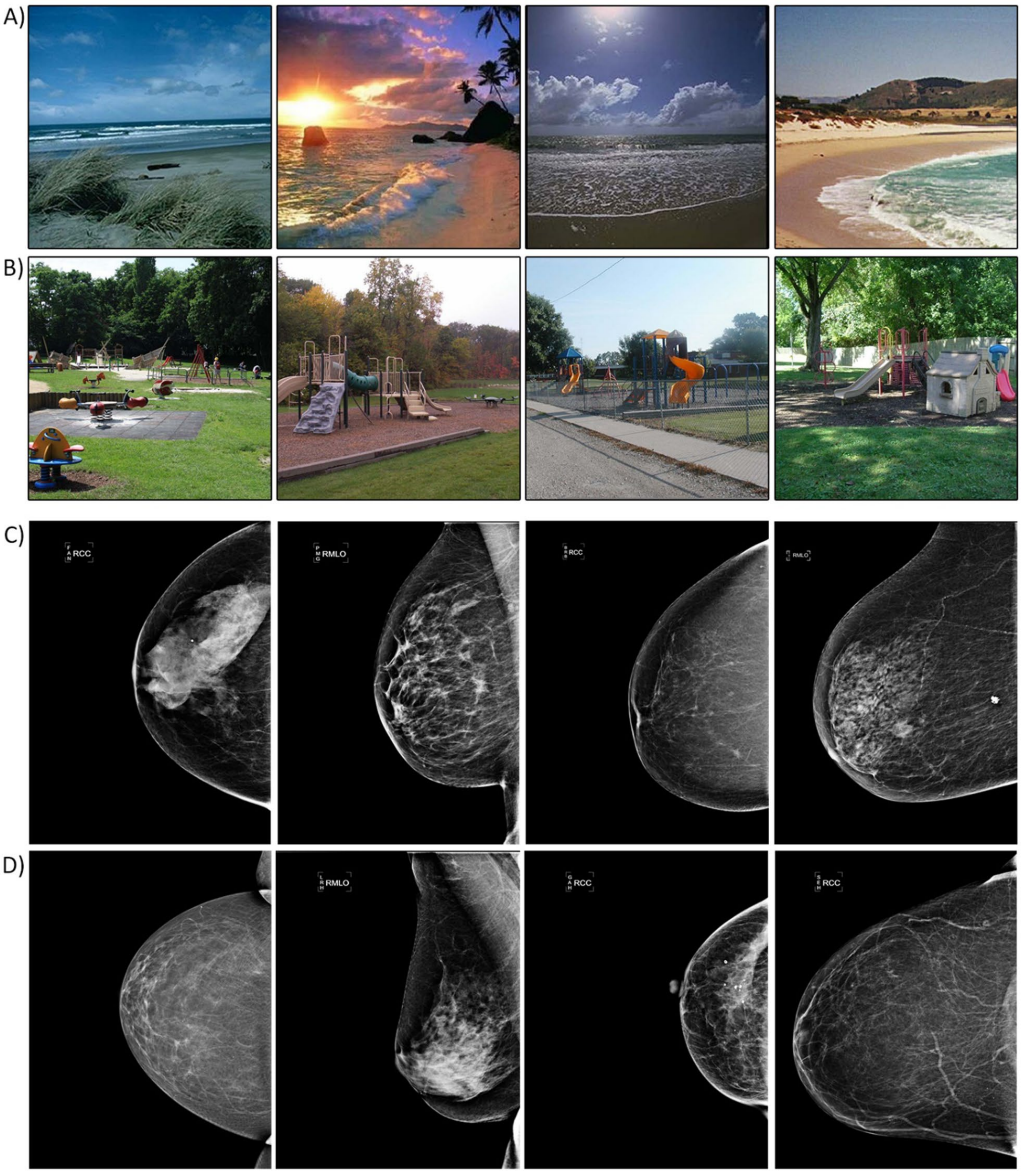
Scene exemplars for beaches (**A**) and playgrounds (**B**) that illustrate the variation in viewing angle, lightning, configuration, and specific objects. Mammogram exemplars containing subtle abnormalities (**C**) or no abnormalities (**D**) illustrating the variation in shape, size, and textural patterns

However, it is not yet known how people acquire these sets of expectations or sensitivity to emergent statistics needed to extract the gist of novel categories, whether that is a natural scene category, or a more abstract categorization of a medical image. Since the learning of natural scene categories happens during normal development, this learning must be able to occur under natural viewing conditions and should not rely on detailed feedback that explicitly explains which features make the scene a beach. Rather, the learning would be expected to reliably occur with broad feedback consisting of just categorical information (‘We are at a beach’). This learning would be in line with the principles of statistical learning, the process through which humans can extract naturally occurring statistical patterns in space and/or time (Turk-Browne et al., 2005).

Indeed, statistical learning leads observers to recognize temporal or spatial statistical regularities and patterns in auditory or visual stimuli after a multitude of exposures without explicit instructions on what to learn (Turk-Browne, 2012). For example, passively viewing a stream of symbols produced strong familiarity feeling for viewed patterns (Fiser & Aslin, 2002a). Interestingly, children as young as 9 months old pay more attention to arrays containing previously seen shape arrangements than new arrangements (Fiser & Aslin, 2002b), suggesting that statistical learning takes place from early on in our development. While the previous examples used simple shapes, statistical learning also extends to more complex stimuli, such as scene images. Observers report more familiarity with scene sequences, such as a kitchen followed by a forest, that were previously seen in a visual stream (300 ms each) without being instructed to pay attention to the order of scene categories (Brady & Oliva, 2007).

Statistical learning is often investigated in the context of temporally separated stimuli, but as previously stated, it also occurs over spatial regularities, which would form the basis for gist category learning. Indeed, observers become familiar with the configurations of complex objects in a grid through repeated exposure (Fiser & Aslin, 2001), and they can decrease their reaction time in a search task due to repeated configurations of distractor arrays without recognition of repeated arrays occurring (Chun & Jiang, 1998), as they implicitly learn to recognize the regularities in contextual cues, or in other words, invariant visual properties, allowing them to interact with the environment more efficiently (Chun, 2000). Similarly, someone might learn to recognize the invariant global properties of a forest, beach, or even an abnormal mammogram through statistical learning of spatial regularities. Statistical learning with global feedback allowed observers to recognize camouflaged objects by learning the general statistics of the background (Chen & Hegdé, 2012). Thus, in our definition of statistical, implicit learning, no assumptions are made about the unconscious nature of the learning or complete lack of awareness of learned patterns, but only that it consists of learning through repeated exposure without explicit instructions or feedback on which features or patterns to extract. We expect that statistical learning through repeated perceptual exposure to novel categories and their group labels would allow observers to acquire the gist of a new category.

To investigate the learning of gist signals, a category is needed in which observers can be trained to improve. Previous training research has shown that the speed of gist extraction from natural scenes is already optimized and at ceiling levels, as extensive training across 15 days did not significantly speed up the reaction time of a 2-AFC animal absent/present task (Fabre-Thorpe et al., 2001). While accuracy increased slightly and average reaction time decreased slightly for familiarized stimuli, this did not transfer to new stimuli and was mostly driven by an increase in speed/accuracy for the most difficult familiarized targets with RTs above 400 ms. Thus, the processes underlying gist extraction for scenes of categories are already highly efficient in adults and do not seem to be able to be further compressed or enhanced. Thus, scenes cannot be used to investigate the processes involved in the learning of a new category of gist. However, it does underline the fact that scene categories must be deeply familiar to the average human observer, which would only be possible if the global gist is learned through the rare instances of explicit feedback (‘these exact features make this a beach/forest/mountain’) or, as we hypothesize, is largely learned through the frequent global feedback moments we encounter in daily life (‘you are in a forest’). Interestingly, expertise within a specific object category, such as cars, will increase the ability to rapidly detect scenes containing that object category, but not others (e.g. humans), in a simultaneous presentation of two scenes (Reeder et al., 2016), adding support to the idea that expertise in a category might influence rapid detection of that category, similar to what is seen in medical experts.

For the gist of medical abnormality, previous research has repeatedly shown that, as expected, naïve observers are unable to extract this signal (Evans et al., 2013a, 2013b; Raat et al., 2021), showing that the general population is not familiar with this gist signal representing a medical abnormality. Interestingly, however, a recent study trained naïve observers to recognize obviously visibly abnormal mammograms (microcalcifications/breast mass) with above-chance accuracy after approximately 600 cases of training (Hegdé, 2020), showing that non-medically trained observers can develop the perceptual ability to recognize obvious abnormalities on free-viewing tasks. This indicates that naïve observers can, at the very least, learn to recognize perceptual characteristics of lesions in mammograms a localized signal, which suggests they might also be able to be trained to recognize the gist signals of abnormality in the overall tissue.

Thus, this study’s aims are twofold: to investigate whether/how people can learn the categorization of a new gist signal (medical abnormality) and to explore which perceptual features in mammograms might drive this gist signal. We will evaluate whether naïve observers can learn to rapidly recognize the gist of a new category after repeated perceptual exposure through training with global feedback, and if this learning is retained after the end of training. Global feedback is defined as the ground truth of the trial, without additional instructions on the location of abnormalities or potential features that might indicate the ground truth. In other words, the task and label are both made explicit, but since no further guidance on which content to use is provided, only implicit/statistical learning can be used. Since the gist of abnormality is a global signal, learning to recognize the gist of abnormality should improve performance on not only mammograms with visible abnormalities, but also on mammograms with only global signals of abnormality, such as contralateral mammograms or those taken prior to the development of localizable cancer, similar to the ability of trained medical experts (Brennan et al., 2018; Evans et al., 2016, 2019). Based on the framework of gist development, and the previous findings of Hegdé (2020), training is expected to induce learning of the gist of medical abnormality, and this is expected to improve performance for mammograms with and without local abnormalities.

As an extension to the training findings, we will also evaluate the performance of a state-of-the-art machine learning model on the same images and compare it to human perception. Human statistical, implicit learning shares key similarities with the concept of deep learning, a computational method where each decision is compared to the feedback of a simple label, inducing learning through backpropagation of the error between the decision and ground truth, which can lead to tuning towards statistical regularities in the input (Voulodimos et al., 2018). Both describe conceptually similar processes that could underlie learning without explicit rules or instructions. As one type of computational modelling, deep learning was developed based on observed brain architecture and processing (Voulodimos et al., 2018). Deep learning models can capture complex visual patterns, allowing for object (Ouyang et al., 2016; Simonyan & Zisserman, 2014) and facial recognition (Taigman et al., 2014).

By comparing human and machine performance on specific images, we can learn more about whether these models capture the same image features that humans might be using—which in turn can be informative for human perception. The single breast classifier (SBC) version of Wu et al. (2019) deep neural network (DNN) for breast cancer screening predicts the probability of both benign and malignant abnormalities for individual unilateral mammograms and reaches a high performance (AUC malignant: 0.84–0.90, AUC benign: 0.74–0.76) on detecting visible abnormalities in a large screening data set, which make it suitable for our purposes. We will use both the SBC and SBC heatmap (SBC + HM) version, which adds heatmaps generated via a secondary network which examines smaller pixel patches for their malignancy probability. These heatmaps provide additional scrutiny of local information that is expected to improve performance, while the SBC without heatmaps would be more equivalent to the global information used in gist extraction. Comparing the probability scores from both the SBC and SBC + HM network to human rating scores will allow us to investigate whether they capture similar information used by human gist extraction of medical abnormality.

## Methods

### Participants

Nineteen adults without previous medical training or experience with viewing mammograms took part in this multi-session experiment, of which 4 withdrew their participation during the training phase. The remaining 15 participants were included in the final data set (aged 20–38, average age 23, 11 female) as they all passed the pre-determined exclusion criteria. Exclusion criteria were predefined in order to exclude participants if there was significant evidence to suggest inattention, defined as (1) having missed more than 30 out of 144 attention trials in total across the 9 training sessions, (2) having failed more than 6 out of 16 attention trials in one training session, or (3) having rated 85% or more of the trials as 50 in any testing session or more than 1 training session. Attention trials which were randomly interspersed across different points in the training sessions briefly showed an image of a beach or forest, which the participant was asked to categorize, a task that should be trivial if the screen was attended.

Participants received a compensation of 50 pounds for their time (~ 5 pound per hour) after completing all 10 sessions and they receive a bonus payment of 10 pounds if they passed 95% or more of the attention checks, as an incentive for them to pay close attention to each trial. Participants all had normal or corrected-to-normal vision. All participants had completed at least their A levels or equivalent. The sample size was based on the work by Hegdé (2020), which reported significant learning during an untimed mammography training experiment with 11 and 14 general population participants in two separate experiments.

### Stimuli and apparatus

The stimuli used in this experiment were 8-bit PNG images of four categories of anonymized unilateral mammograms in mediolateral oblique (MLO) or craniocaudal (CC) view: normal mammograms of healthy women (normal), mammograms with obvious cancerous abnormalities (obvious), mammograms with subtle cancerous abnormalities such as architectural distortions (subtle), mammograms without visibly actionable lesions that are thought to contain global features of abnormality (either contralateral to a breast with a cancerous abnormality (contralateral), or mammograms taken 1 to 6 years prior to visible actionable sign of abnormality appearing in a subsequent scan (priors)). The labels ‘obvious’ and ‘subtle’ were categorized as such by an experienced radiologist for the Complex Cognitive Processing Laboratory of the University of York. Further information about cancer-type descriptors can be found in Appendix 1. Contralateral and prior cases were combined into one category, as both contain global signals of abnormality and lack any localizable lesions. The normal, obvious, subtle, and contralateral cases were sourced from the OPTIMAM database. The priors were sourced from the Complex Cognitive Processing Lab database in collaboration with Dr. Bradley of the York Hospital for this study. The majority of selected mammograms were acquired with Lorad Selenia (75.4%) and Selenia Dimensions (13.5%), with a smaller portion of mammograms acquired with Senographe Essential (8.9%) and the L30 (1.8%), and a minority taken by MammoDiagnost DR (0.3%) and Mammomat Novation DR (0.1%). All mammograms that are part of the Complex Cognitive Processing Lab database of stimuli can be shared with other researchers upon reasonable request to the senior author (K.K. Evans), while the OPTIMAM database is also available for research purposes through an application process (https://medphys.royalsurrey.nhs.uk/omidb/getting-access/).

The training set was composed of 5668 unilateral mammograms, consisting of 1558 normal, 1019 obvious, 899 subtle, and 2192 global (1868 contralateral, 324 prior) images, so approximately 72% of the available stimuli contained the gist of abnormality. This large data set ensured that participants were trained on a wide range of mammograms and reduced the number of repetitions. Some repetitions occurred randomly across the 36 blocks, but never within a block: on average, normal mammograms were repeated 0.9 times, obvious, subtle, and contralateral mammograms were repeated < 0.1 times, and priors were repeated 2 times.

The testing set consisted of 200 unilateral mammograms: 80 normal, 30 obvious, 30 subtle, 30 contralateral, and 30 prior mammograms, meaning 60% of the stimuli contained the gist of abnormality signals. The same images were used for each test session to equate the difficulty level across participants and testing phases, and these were not used during training phases. Previous research has shown very low recognition memory in both general population (d’ prime = 0.36) and radiologists (d’ = 0.86) when tested on recognition directly after exposure to 72 mammograms viewed for 3 s each (Evans et al., 2011a, 2011b). Since we use a larger number of mammograms shown for shorter durations and with longer inter-exposure intervals no significant memory effects were expected, especially since no feedback was given on the test cases.

To further characterize the test cases, an experienced mammogram reading radiologist assessed each mammogram on radiological perceptual features. The following radiological features were rated: 1) four-point BIRAD breast density scale (D’Orsi et al., 2018) as (I) fatty, (II) mixed but predominantly fatty, (III) mixed but predominantly glandular, and IV) extremely dense), (2) breast pattern as normal or complex, and (3) level of concern/suspicion on a five-point scale from (I) normal, (II) benign, (III) indeterminate, (IV) suspicious, and (V) malignant. Chi-square tests of independence showed no significant association between density and image type (*Χ*^2^(12) = 9.63, *p* = 0.648). Associations between image type and both breast pattern (*Χ*^2^(4) = 11.50, *p* = 0.021) and level of concern (*Χ*^2^(16) = 138.05 *p* < 0.001) underline that an experienced radiologist could detect radiological perceptual differences in our cases, but that these signals were not driven by density. Thus, simply becoming sensitive to the density of mammograms would not result in significant increases in performance. This is in line with previous studies that also showed a lack of correlation between BIRAD density and gist of abnormality ratings (Evans et al., 2013a, 2013b, 2016, 2019).

The experiment took place on a computer or laptop screen, with the participant using a mouse and keyboard to submit rating responses. Since the experiment took place online, the exact apparatus varied between participants. However, physical stimulus size was equated by using a screen calibration method using either diagonal screen length or a credit card size matching task inspired by the method proposed by Li et al. (2020) to ensure the images were displayed as 12.8 cm/5 inches high by 15.75 cm/6.2 inches wide across all sessions and participants. The experiment was accessed via a website optimized for Firefox and Chrome browsers, where participants could log in for each session according to the scheduling rules, using their unique user ID.

### Procedure

**Fig. 2:**
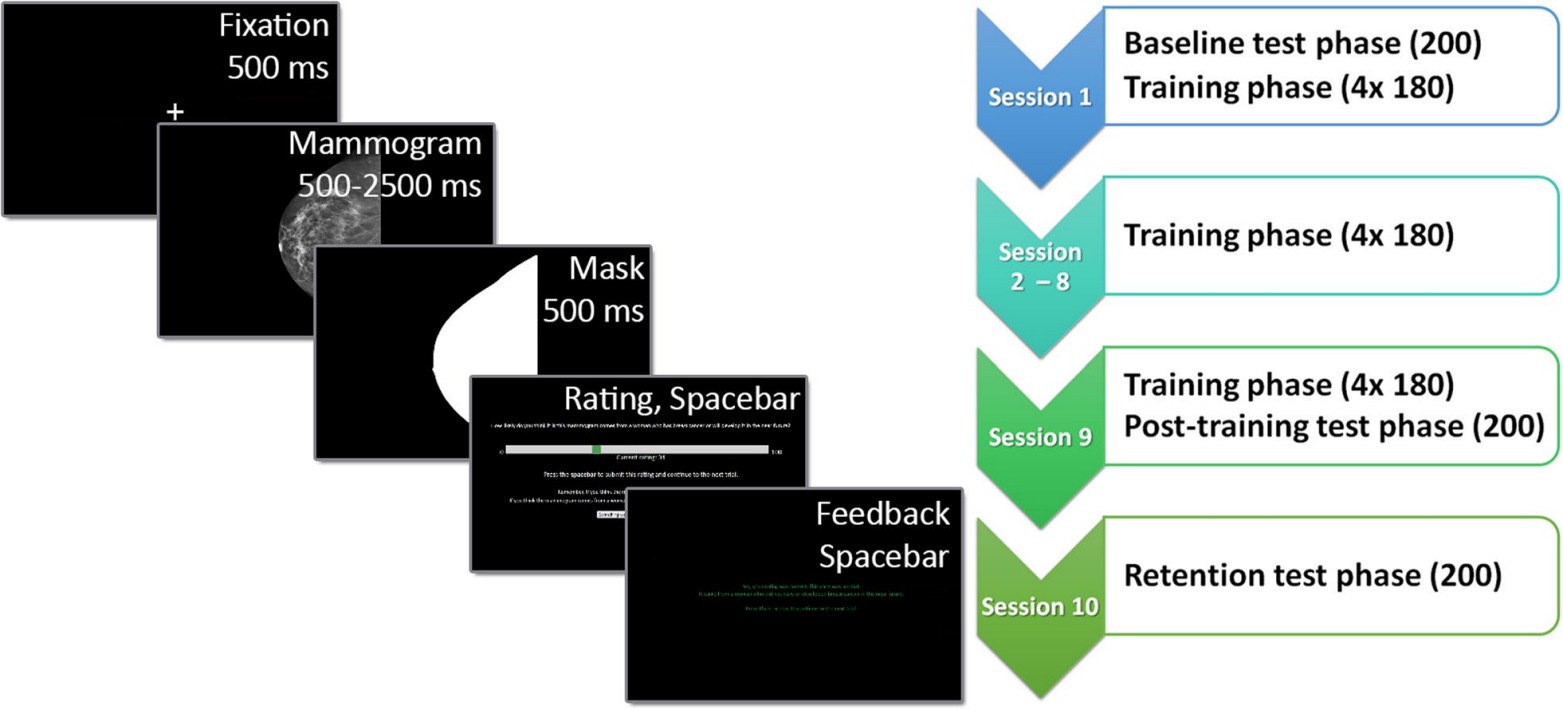
Overview of the experimental procedure and flow chart schedule of the experiment. The screens show the presentation order within a training trial and the duration or button press to continue. Test trials always showed mammograms for 500 ms and omitted the feedback screen but were otherwise identical. The flow chart schedule shows the order of experimental phases for each session, and the number of unilateral mammograms viewed per session. In the test phases, 200 mammograms were viewed, while the training phases had 4 blocks with 180 mammograms each. Sessions 1 to 9 were separated by 1 to 3 days each, while session 10 was delayed by 7 to 10 days after session 9

This study used a multi-session within-subject repeated measures design. It consisted of a total of 9 training phases and three testing phases completed across 10 sessions spread out over multiple days, as is summarized in the flow chart of Fig. 2. Before the first session, each participant joined an individual video conferencing call via Zoom with the experimenter to guide them through the instructions and check for any questions or technical difficulties. During this conference call, the participants also watched a pre-recorded instruction video, explaining what a mammogram is and what the experiment task is, to ensure all participants received identical instructions. The first session started with a pre-training test phase to establish a baseline of performance. After the pre-training baseline, participants immediately performed the first training phase, which was followed by 7 subsequent sessions consisting of a training phase each, separated by at least 1 and at most 3 days each. The 9th session consisted of the last training phase and a subsequent post-training test phase to measure potential improvements in performance. The tenth and last session took place 7 to 10 days after the last training session and consisted of a retention test of performance. Participants scheduled their own sessions according to these scheduling rules but received regular reminder emails to inform them when their next session was due.

Both test and training trials followed a similar format (Fig. 2). They each consisted of a fixation cross (500 ms), the mammogram (500 ms or 500–2500 ms), a mask of the filled shape of the mammogram (500 ms), followed by a rating scale between 0 and 100 (self-paced). Participants were asked to give their decision by adjusting a cursor on a rating scale that would indicate how sure they were that a unilateral mammogram was normal or abnormal. This rating was then used as a performance measure applying signal detection theory methodology. In the training trials, this was followed by a feedback screen (self-paced). Feedback was based on the rating decision and ground truth, e.g. if the ground truth was abnormal, ratings above 50 were counted as correct, and ratings of 50 or below were counted as incorrect. The feedback screen informed participants whether their submitted rating was correct or incorrect, and whether the ground truth for the trial was normal or abnormal. The colour of the text was green for correct and red for incorrect ratings. Participants received no feedback during the test phases.

Each test phase consisted of 203 trials: three practice trials with feedback to familiarize them with the task, then 200 test trials showing the pre-selected test set in a randomized order. The test set consisted of 80 normal mammograms, and 30 each of the four abnormal categories (see stimuli and apparatus for more details). Each mammogram was shown for 500 ms before the mask and then the rating screen appeared.

Each training phase consisted of a total of 736 trials, split into 4 blocks of 184 trials each: 180 mammograms, and 4 attention trials dispersed throughout each quarter of the block. The 180 mammograms were randomly selected from the training set to show 72 normal mammograms, 27 obvious, 27 subtle, and 54 global abnormal mammograms. More global abnormal mammograms were shown because these are thought to be both the most difficult, and the most likely to contain the global gist signal, on which we would expect increased performance if indeed a gist signal was learned. The attention trials showed easily recognizable colour photographs of either a forest or a beach and had an alternative rating instruction to rate beaches as 0 and forests as 100. These trials also showed feedback based on the response; however, if the answer was incorrect, the feedback screen was shown for at least 10 s before they could continue, and the attention trial was repeated until they answered correctly. Participants were encouraged to take self-paced breaks in between each block.

During the training session, the maximum viewing time for the mammogram started at 2500 ms in the first block to familiarize the participants with the procedure and task. Participants were encouraged to press the spacebar as soon as they had a first impression to continue to the mask and then the rating screen (minimum viewing time 500 ms). However, this was not required, and the mammogram would automatically be replaced by the mask at the maximum viewing time. In subsequent blocks, maximum viewing time was adapted based on performance: if the total d’ prime for the block was above 0.2, max viewing time was decreased to 90% of the average actual viewing time of that block, but if d’ prime was below 0.05, it instead increased to 105% of the current maximum viewing time to a maximum of 2500 ms.

### Data analysis

Signal detection measures were used for analysing observers’ performance, as these can differentiate performance (d’) and response biases (criterion) in a binary classification task, calculated from the proportions of hits and false alarms. D’ characterizes the accuracy of performance, with a d’ of 0 representing chance and higher values representing better performance. Criterion characterizes response bias, with a criterion of 0 being unbiased, a negative criterion is liberal, meaning that in any random trial the participant is more likely to label it as abnormal than normal, and the opposite is true for a positive criterion, which is conservative, leaning towards rating trials as normal.

First, the proportions of hits and false alarms were calculated from the rating and ground truth (normal or abnormal) of the trials for each mammogram category. The numerical rating for a trial was compared to the set threshold of 50 for d’ and criterion: the binary rating decision was considered ‘normal’ if below, or ‘abnormal’ if above the threshold. D’ was then calculated by subtracting the z-transformed false alarms from the hits (d’ = z(hits)−z(false alarms)). A d’ of zero represents chance performance, with positive values representing above-chance accuracy. Criterion on the other hand adds the z-transformed hit and false alarm rates and divides them by − 2 (c = (z(hits) + z(false alarms))/−2). As the task explicitly instructed participants to rate normal trials below 50 and abnormal trials above 50, and to rate more extreme values the more confident they were, d’ and criterion at threshold 50 were the primary outcome measures of performance.

To further characterize the shape of the rating curves at different points of the experiment, area under the curve (AUC) measurements of receiver operating characteristic (ROC) curves were used. ROCs were constructed by repeating the division of trials into proportions of hits and false alarms using a sliding value of normal/abnormal rating thresholds (1–99) and plotting all data points, from which the AUC was then calculated in Python. AUC ranges from 0 to 1 and represents the probability that a randomly chosen abnormal trial will be rated higher than a randomly chosen normal trial (Hanley & McNeil, 1982), with chance performance in a raw rating experiment yielding an AUC of 0.5 and higher AUCs representing more accurate performance.

The average and median viewing time of different screens were also calculated for the mammogram screen (training phases only), rating screen, and feedback screen (training phases only) for each of the sessions. Outlier rating times (outside of mean plus/minus 3 STD of the individual session) were excluded.

The main research question of whether naïve observers can learn a new category of gist through perceptual training was evaluated using 3-by-3 two-way repeated measures ANOVAs with 2 factors: testing moment (3 levels, pretest, post-test, and retention test), and image type (3 levels, obvious, subtle, global) for d’ prime and criterion. To evaluate whether participants were engaged with the task, attention checks and feedback viewing time were evaluated with descriptive statistics. Additionally, to investigate potential differences in rating speed, which might signify elements of decision-making speed, before and after training, a 4-by-3 two-way repeated measures ANOVA was performed on rating time across the testing sessions (pre, post, retention) and image types (normal, obvious, subtle, global). For any repeated measures ANOVA with a significant effect of testing moment, planned simple contrasts were performed comparing the pretest and post-test, and the pretest and retention test, as this was the primary research interest. Pearson’s correlations were calculated for d’ across the training phases, to evaluate whether individual performance improved throughout the training period. Based on the correlation coefficient, participants could be divided into learners (above 0 coefficient) and non-learners (below 0 coefficient), which were investigated with the main aim to explore the main effect of testing phase on performance. This method was also used on a bootstrapped simulation of a population making random rating decisions, to ensure that any learner vs non-learner effects were not caused by chance.

As an additional means of assessing whether participants outperformed chance, alternative log-linear-likelihood ROCs and AUCs were calculated and compared to chance levels. This was based on the methodology suggested by Semizer et al. (2018) to handle potential bimodal distributions that can result from raw rating experiments more accurately. ROC curves were smoothed with a Gaussian kernel, width 10, after which log-likelihood ratios were calculated to compute the area under the curve (AUC). ROC curves and their AUCs are calculated for the real data and 100 randomly bootstrapped samples (with resampling). If the AUC of the real ROC was higher than the 95^th^ percentile of the randomly bootstrapped AUCs, this strongly suggests that the participant outperformed chance.

Lastly, as exploratory analysis, we compared the ratings by human observers to the probability scores of benign/malignant findings from a deep neural network (DNN). Single unilateral mammograms were evaluated using the single breast classifier (SBC) and SBC plus heatmap (SBC + HM) version of Wu et al. (2019) DNN for breast cancer screening. 16-bit PNG versions of each unilateral mammogram were pre-processed to remove annotations and then run through the SBC and the SBC + HM. DNN inference was accomplished on Cloud Viking, a University of York HPC cluster. The compute nodes used were equipped with a NVIDIA V100 GPU. Stimuli supplied to the SBC had higher pixel dimensions than those shown to human observers, and a greater bit-depth, due to the requirements of the SBC. The output consisted of prediction scores for benign and malignant findings for each mammogram, ranging from 0 to 1, which were transformed to 0-to-100 scale to match the human rating scale. AUCs were calculated for the SBC and SBC + HM to evaluate overall performance. Image-level and category-level comparisons between human and SBC scores were made using Spearman’s rank correlations, to investigate the level of agreement. These correlations were compared before and after training, to see if training increased the level of agreement between human and machine scores.

## Results and discussion

### Human observer performance in training to detect cancer

#### Attention and task engagement

Participants were highly attentive during the training phases, as indicated by the very low number of incorrectly answered attention check trials (median 0, mean 0.93, std 1.24, max 4) across the 144 total checks in the 9 training phases. Additionally, participants actively used the spacebar to continue to the rating screen, meaning that both their average and maximum viewing time rapidly decreased from 2500 ms, with all participants showing below 600 ms average maximum viewing time during the fourth training phase (see Appendix 2 for more details on engagement and viewing times).

#### Effect of training on performance measures

**Fig. 3:**
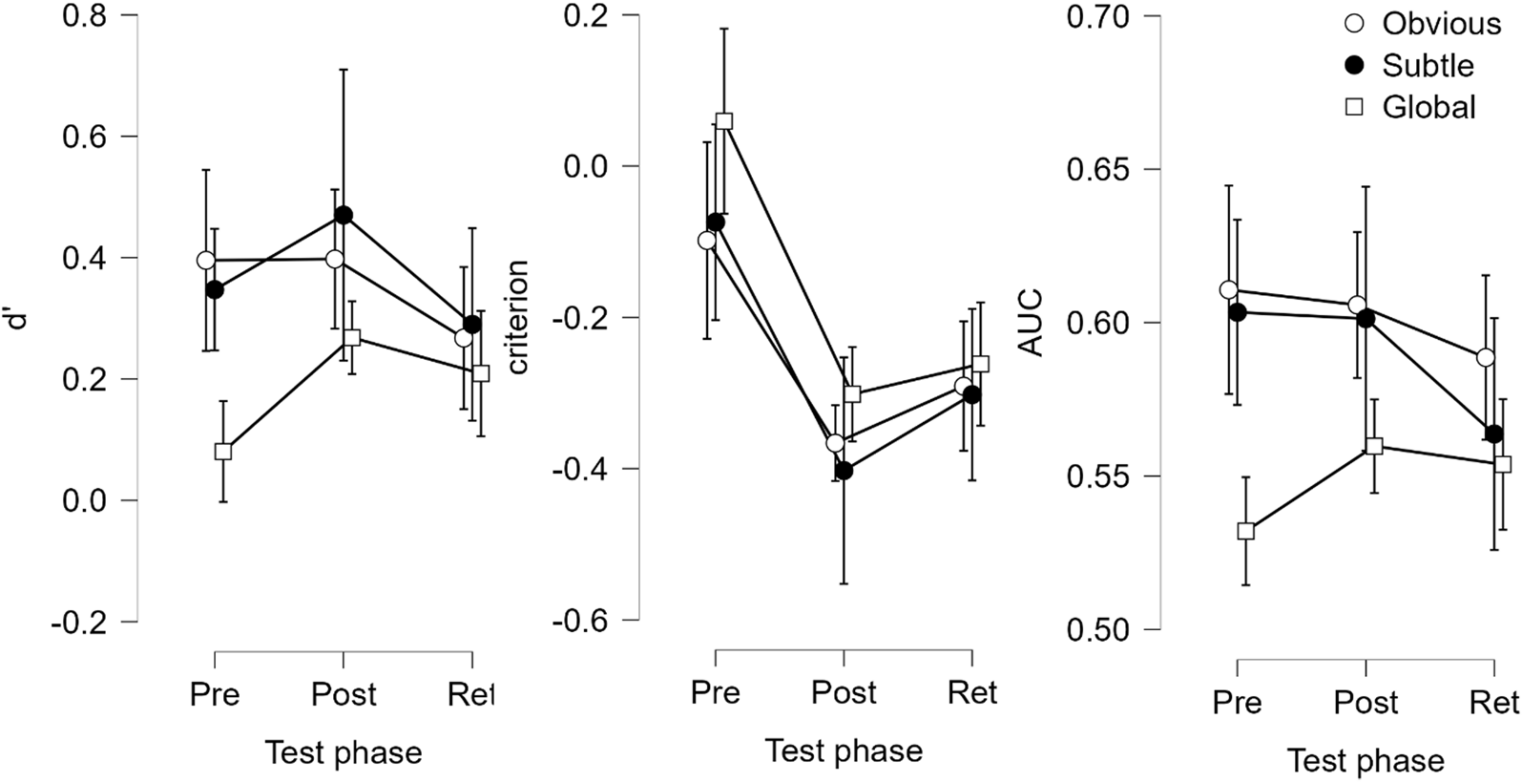
Mean d’, criterion, and AUC across test phases (± 95% confidence intervals) for all participants (*n* = 15), plotted separately for each abnormal image type (‘circle’ Obvious, ‘Bullet’ Subtle, ‘Square’ Global)

Figure 3 shows the mean d’, criterion, and AUC for each image type pre-training, post-training, and at retention. Averaged over image types, d’ increased after training in 12 out of 15 participants, with a mean d’ of 0.274 ± 0.058 prior to and 0.378 ± 0.079 after training, and 0.255 ± 0.086 at retention. Compared to pre-training, rating criterion became more liberal after training in 14 participants, and remained more liberal at retention in 13, with a mean criterion of − 0.0377 ± 0.073 prior to, − 0.356 ± 0.112 after training, and − 0.284 ± 0.114 at retention. Meanwhile, AUC was higher than pre-training in 9 out of 15 after training, and in 6 out of 15 at retention, with a mean of 0.582 ± 0.016 prior to, 0.589 ± 0.016 after training, and 0.568 ± 0.018 at retention. Similarly, log-linear-likelihood AUCs were compared to bootstrapped chance levels, which showed a sizeable increase in participants performing above-chance levels after training (see Appendix 3). Additionally, analysis of average and median rating times showed that participants took significantly less time to make rating decisions after completing their training (see Appendix 4).

3 × 3 repeated measures ANOVAs with the factors testing phase (pre, post, retention) and image type (obvious, subtle, global) were used to investigate the effect of training on d’, AUC, and criterion. For d’, this showed evidence of an image type effect (*F*(1.433,20.066) = 7.451, *p* = 0.007, ηp^2^ = 0.347 with Greenhouse–Geisser correction), while the testing phase effect was trending towards significance (*F*(2,28) = 2.816, *p* = 0.077, ηp^2^ = 0.167) and there was no significant evidence for an interaction effect (*F*(4,56) = 1.455, *p* = 0.288, ηp^2^ = 0.094). The image type effect was also observed for AUC (*F*(1.292,18.088) = 11.242, *p* = 0.002, ηp^2^ = 0.445), while there was no significant evidence for a testing phase (*F*(2,28) = 1.191, *p* = 0.319, ηp^2^ = 0.078) nor interaction effect (*F*(4,56) = 2.005, *p* = 0.106, ηp^2^ = 0.125). However, AUC was seen as less informative than d’ in this experiment, as participants were explicitly instructed to rate trials below 50 for normal and above 50 for abnormal decisions, meaning the cut-off was fixed. Overall, there was no significant evidence of improvements as a result of training, but the trending *p* value for d’ suggests this might be due to individual variation in learning ability in the testing group, which will be further explored in the following section on performance throughout training.

On the other hand, for criterion, the 3 × 3 RM-ANOVA showed a significant effect of image type (F(1.433,20.066) = 7.451, *p* = 0.003, ηp^2^ = 0.347 with Greenhouse–Geisser correction) and of testing phase (*F*(1.352,18.922) = 11.501, *p* < 0.001, ηp^2^ = 0.451 with Greenhouse–Geisser correction), but no evidence for an interaction effect (*F*(4,56) = 1.455, *p* = 0.228, ηp^2^ = 0.094). Overall, the criterion differed significantly between baseline and both post-training (Estimate: − 0.319, *t*(28) =  − 4.571, *p* < 0.001) and retention (Estimate: − 0.247, t(28) =  − 3.542, *p* = 0.001). In summary, perceptual training made participants more likely to rate any given trial as abnormal. This could indicate that participants tended to put more weight on negative feedback when they missed a cancerous case than when they incorrectly labelled a normal case as abnormal, causing a shift towards liberal rating bias. Importantly, however, participants were not instructed to preferentially avoid one type of error over the other.

#### Performance throughout training

**Fig. 4:**
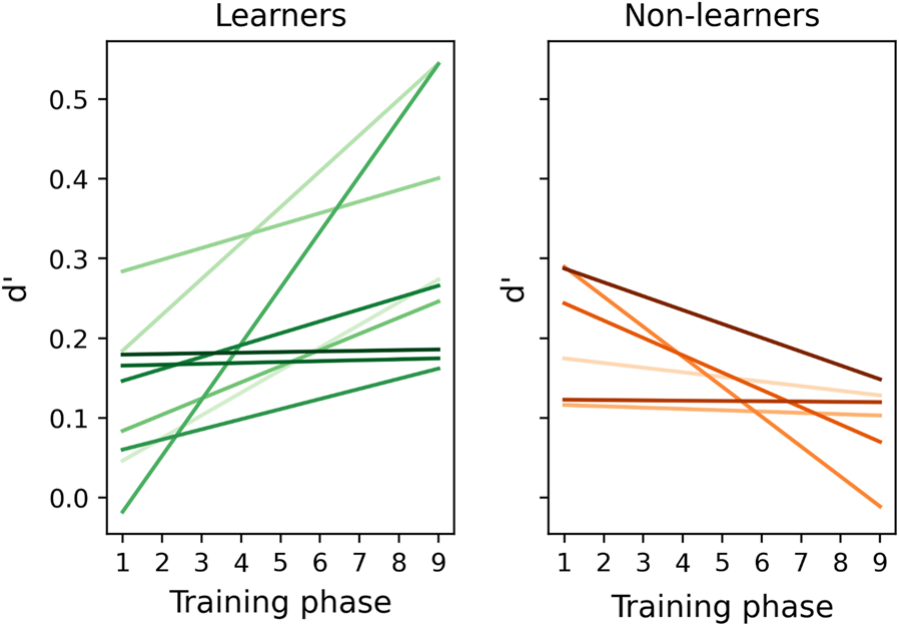
Individual progression of d’ across the 9 training phases, with the learners in green hues in the left plot and the non-learners in orange hues in the right plot

To investigate performance improvements across training phases, linear Pearson’s correlations were calculated between d’ across image types and training phase, numbered 1 through 9 (Fig. 4). Correlation coefficient varied considerably across participants, with an average of 0.109 ± 0.239. Notably, a positive correlation was found between d’ and training phase for 9 participants (average 0.418 ± 0.172) and a negative correlation of the remaining 6 (average − 0.357 ± 0.245). This indicated that in the training groups there might be learners and non-learners when dividing participants based on their ability to improve their performance on this specific perceptual learning task.

**Fig. 5:**
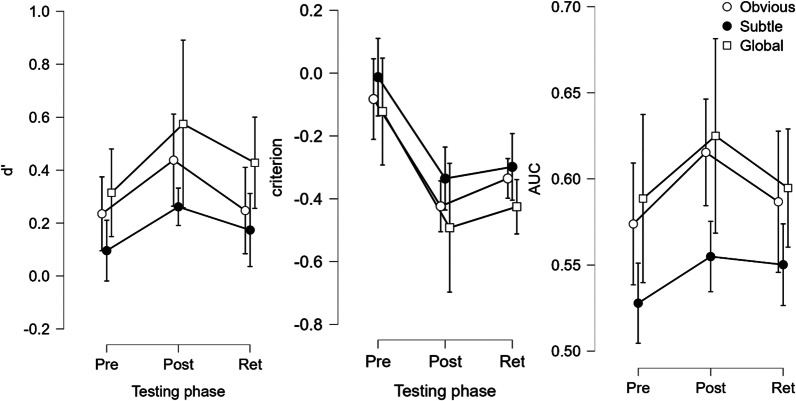
Mean d’, criterion, and AUC across test phases (± 95% confidence intervals) for the learners (*n* = 9), plotted separately for each abnormal image type (‘circle’ Obvious, ‘Bullet’ Subtle, ‘Square’ Global)

To further explore this, analysis of performance measured by d’ was repeated separately for learners and non-learners, to see if the learning during the training phases translated to improved performance on the test phases. For learners, it showed that d’ was affected by both image type (F(2, 16) = 13.169, *p* < 0.001, ηp^2^ = 0.622) and testing phase (F(2,16) = 4.597, *p* = 0.026, ηp^2^ = 0.365), without interaction effect (F(4,32) = 0.223, *p* = 0.924, ηp^2^ = 0.027). Planned comparisons for the testing phase effect with a simple contrast showed that post-training d’ was significantly higher than pre-training levels (difference: 0.209, t(16) = 2.971, *p* = 0.009), while this was not the case at retention (difference: 0.068, t(16) = 0.962, *p* = 0.350) (see Fig. 5). On the other hand, for non-learners, d’ was not significantly affected by image type (F(1.091, 5.455) = 3.409, *p* = 0.118, ηp^2^ = 0.405) or testing phase (F(2,10) = 2.184, *p* = 0.163, ηp^2^ = 0.304), but did show evidence for an interaction effect (F(4,20) = 4.254, *p* = 0.012, ηp^2^ = 0.460). Post hoc comparisons for this interaction effect with Holm correction showed that this was driven by significant differences between obvious and subtle pre-training (d’ difference: 0.579, t = 4.438, *p* = 0.005), and between obvious pre-training and global at retention (d’ difference:4.165, t = 4.165, *p* = 0.008), both of which do not signify learning of the gist signal. Thus, for learners, d’ improved significantly after training and returned towards baseline levels at retention, suggesting that the learning period was not sufficient for long-term retention. The fact that these effects were not found for the non-learners suggests there is individual variation in people’s ability to obtain the gist of a new category through this type of online training. Analyses of the criterion can be found in Appendix 5.

These results were compared to those expected under random chance to further ascertain that the split in learning effect was caused by individual differences, rather than any selection bias caused by applying a criterion based on Pearson’s correlation coefficients. Random rating decisions were simulated across 1000 runs of 15 participants each, calculating their performance on the pre-training and post-training test phase, and each of the 9 training phases, and splitting them into learner and non-learner categories with the same Pearson’s correlations as used for the real observers. The difference between pre- and post-training d’ for ‘learners’ was on average 0.001 ± 0.006, while for the ‘non-learners’ this was 0.002 ± 0.006 (95%CI). This clear lack of improvement in both simulated groups demonstrates that the observed split in learners and non-learners cannot be explained by random effects.

Our results show that nine sessions of perceptual training with global feedback were sufficient to induce a small, but robust increase in gist recognition across all mammogram categories that was significant in the subset of learners. Importantly, this included mammograms that did not contain any localizable lesions, as they were contralateral or prior to the development of a visible lesion, supporting the notion that this was a global signal, and not only the local signal that was captured by the learners. Thus, perceptual exposure paired with global feedback was sufficient to learn the gist of a new category in a group of learners.

However, performance returned towards baseline levels after 7 to 10 days of retention without exposure to mammograms, indicating that the learned signal is poorly retained. While this in itself might seem unfortunate, it is evidence that participants underwent perceptual learning of the global gist signal rather than following any rating strategy based on simpler specific local features, as a strategy would be expected to be retained. Instead, this ‘use it or lose it’ aspect fits with the view of perceptual tuning of the visual system to regularly occurring image statistics in the mammogram texture that must be actively maintained. This finding also converges with findings that radiologists’ gist performance correlates with cases reviewed in a year, not years of experience (Evans et al., 2019). Thus, showing it is recent, continued perceptual experience, and not only (medical) knowledge that allows gist extraction to occur.

Further underlining the importance of perceptual experience rather than knowledge for detection tasks is previous research that showed that pigeons could be trained to recognize cancer-relevant microcalcifications in small patches with above-chance accuracy (Levenson et al., 2015). The findings give supporting evidence that mammograms contain perceptual features that can be learned through global feedback in implicit learning. However, importantly, the pigeons could not learn to differentiate benign from suspicious masses nor could they detect cancer before the onset of any visibly actionable lesions, suggesting a limitation of their perceptual capabilities. Thus, while pigeons could potentially be used as a cost-effective medical image observer to, for example, investigate the impact of technical aspects such as spatial frequency, colour, or other display parameters on performance, as suggested by Levenson et al. (2015), our research instead suggests that training naïve human observers might be a more viable alternative, especially for more complex medical imaging categorization tasks, as humans can learn a complex gist of abnormality, and are arguably easier to instruct.

Our findings suggest an important role for individual differences in the ability of a participant to learn the gist of abnormality, resulting in a group of learners and non-learners. This can be compared to the variability in gist extraction performance between individual radiologists, which partially but not fully correlates with recent perceptual exposure, suggesting there are additional individual factors influencing radiologist performance. What’s more, while the learner and non-learner groups were identified based on their learning rate across the nine training phases, further investigation showed that the learner group had an above-chance performance on identifying global abnormalities even before any training had taken place. This is striking, as no local abnormalities are present in these mammograms. Thus, learner participants might already have been more sensitive to disruption of image statistic regularities pre-training than their non-learner counterparts. The previous literature contains numerous examples of individual differences in perceptual sensitivity. Individual differences in performance or sensitivity have been reported across many perceptual domains: in visual search tasks (Brock, Xu, & Brooks, 2011; Sobel, Gerrie, Poole, & Kane, 2007; Wang, Lin, & Drury, 1997), face processing (White & Burton, 2022), scene processing (Pringle et al., 2004), or even low-level visual properties such as colour sensitivity (Emery & Webster, 2019), or auditory temporal processing (Shinn-Cunningham et al., 2017). In this context, it is not surprising that our participants also showed a range of initial sensitivity to the task.

Furthermore, the observed variability in learning rates between participants in this study matches the previous literature. Learning rates differ significantly between individuals across seven perceptual tasks in the visual and auditory domain, such as Vernier acuity, face view discrimination, and auditory frequency discrimination (Yang et al., 2020). Importantly, the contribution of participant-specific (36.8%) factors is approximately equal to the task-specific (~ 38.6%) factors influencing learning rate, underlining the large impact individual differences can have on learning rates across tasks. Individual differences in learning rates have also been demonstrated in spatial learning in virtual environments (Waller, 2000).

So, learners might have been predisposed to have enhanced sensitivity to structural regularities, resulting in above-chance pre-training performance, and subsequently further improved their performance after training. This predisposition might be innate, or due to previous experiences. Innate factors can influence performance and learning, as shown by positive correlations between learning rates and cortical thickness in the posterior parietal cortex (PPC) and motion-sensitive area MT + of the V5 for a motion discrimination visual search task (Frank et al., 2016), and similarly for the left fusiform face area in a face view discrimination task (Bi et al., 2014). Furthermore, previous experiences such as gaming activity might influence brain plasticity and increase general perceptual learning ability (Bavelier et al., 2012; Bejjanki et al., 2014). Another factor that might have made learners more likely to learn the gist signal could be differences in strategy. It is possible learners were tuned to a more global strategy compared to non-learners who might have focused more on local signals. Previous research suggested that learners and non-learner groups utilized different strategies while being trained on a difficult grating orientation task (Dobres & Seitz, 2010). Further research could further explore differences in initial sensitivity, neural markers, and strategies employed by learners and non-learners in a gist learning task.

The fact that non-learners did not show improvement in their ability to detect the gist of abnormality might also be related to the duration of training. Perhaps, these non-learners would have shown improvement after additional training sessions, where this was not the case after nine sessions, for example, due to a slower learning rate or an initial maladaptive learning strategy. Interestingly, in Hegdé’s (2020) design participants trained until a predefined performance level, which took anywhere between 288 to 936 trials, a factor of 3.25 difference, providing evidence for the existence of a range in individual learning times. However, they also reported that 4 participants left part-way through the experiment, leaving it up to question if/when these participants would have reached the predefined performance level. Thus, while non-learners in the current study might have lacked the aptitude or capacity to learn the new gist category in the task format, they might have simply required further perceptual training before they would have been able to increase their performance. Future research could employ a predefined performance threshold similar to Hegdé’s (2020) design to gain further insight into the variation in perceptual exposure needed to learn the gist of a new category.

As briefly discussed above, our results corroborate the main findings of a previous training study that showed that implicit learning through auditory global feedback could induce learning of visual patterns of medical abnormality in a free-viewing task (Hegdé, 2020). Notably, however, the learning described by Hegdé occurred much faster, after an average of ~ 600 trials, and resulted in a higher performance of d’ 2.5. One factor that might explain the difference in performance is the differences between the stimuli. The abnormal mammogram cases used by Hegdé and colleagues contained localizable, and obvious abnormalities with one region of interest at least 200 pixels wide, whereas the current study used a larger variety of mammograms, containing obvious or subtle abnormalities, or even only global signals of abnormalities with no visible lesions. Another factor is likely the difference in tasks, as free-viewing tasks are generally easier than rapid gist extraction tasks. The same effect can be observed for medical experts, as their performance in laboratory free-viewing experiments reached d’ of 2.5 for chest radiographs (Kundel & Nodine, 1975), and d’ of 1.9 for mammograms (Evans et al., 2013a, 2013b), whereas gist extraction performance reached a d’ of 1 for chest radiographs (Kundel & Nodine, 1975), and a d’ of 1 (250 ms) and 1.14 (500 ms) for mammograms (Evans, Georgian-Smith, et al., 2013a, 2013b). Thus, while the current performance did not reach the same levels as observed by Hegdé, this can be explained by differences in task and stimuli.

A general limitation of the current study was the duration of the perceptual training. This had to be limited for the viability of the research, but consequently, naïve participants did not reach the same performance levels as expert radiologists. After training, learners reached an overall average d’ of 0.43, which is close to a medium effect size. Learners did not quite reach the d’ of 0.88–1.14 reported for expert radiologists on obvious/subtle lesions in similar experiments (Evans et al., 2016, 2019; Evans, Georgian-Smith, et al., 2013a, 2013b), but learners’ post-training performance on mammograms with global abnormalities (d’ 0.57) was remarkably similar to the performance of expert radiologists on comparable cases in different experiments, such as a reported d’ of 0.59 on contralateral mammograms (Evans et al., 2016) and a d’ of 0.21 on priors (Evans et al., 2019), demonstrating the validity of the learning. The difference in performance on visible actionable lesions difference could be partially the result of specific medical knowledge, or it could reflect the differences in the magnitude and duration of perceptual training. While medical experts do not routinely perform gist rating tasks, they have years of real-world exposure to the stimuli with an average of up to 4000 read mammograms a year in which they focus on detecting visible abnormalities, which would involve an early non-selective stage of visual processing shaping their knowledge of the gist of abnormality.

In the current study, participants became significantly more liberal in their ratings after training, meaning they were more likely to label any given mammogram as abnormal than before. This could potentially reflect a self-imposed criterion in which participants tried to avoid missing any cancerous cases at the cost of more false alarms—although it is important to note that no such instruction was given in the experiment. A move to a more liberal decision criterion may indicate the participants’ feeling of familiarity with images after training and thus more willingness to report a signal but it is more likely a result of early stages of learning-related changes in developing perceptual expertise as observed in some perceptual training studies (Aberg & Herzog, 2012; Palmeri et al., 2004; Xu et al., 2016).

Another interesting observation was the change in rating time, as participants became significantly faster after training. This increase in rating speed could potentially be a marker of the development of expertise. Decreases in reaction times have previously been described to occur in naïve learning to categorize aerial photographs (Lloyd et al., 2002) and training on face-like artificial object categorization (Wong et al., 2009). However, other studies reported no consistent changes in reaction time after training subordinate and superordinate level bird categorization (Devillez et al., 2019; Jones et al., 2020). Additionally, the interpretation of our findings is complicated by the fact that this study used a 0–100 rating scale, operated using a mouse. Thus, it is also possible that participants habituated to using the slider and became faster at reaching their desired rating score. Overall, this increase in rating speed is an interesting observation, but a different design is needed to be certain that this effect is caused by changes in decision-making time rather than adeptness at the rating task.

### Deep neural network performance in detecting cancer

With the aim of further understanding how gist expertise develops we examined whether a DNN, analogous to human implicit learning, was able to capture the same image statistics that humans might be using when learning to detect the gist of the abnormal. We use a DNN specifically developed for malignancy detection, which was pre-trained on mammograms, to evaluate its performance on the mammograms we used for training and testing our human learners. This is assessed using the DNN’s calculated malignancy probability scores (Wu et al., 2019), the probability that that mammogram contained a malignant abnormality. Each unilateral mammogram in the training image set and test image set was scored by both the single breast classifier image-only (SBC) and SBC + heatmaps (SBC + HM) DNN. The DNN also provided benign probability scores, the probability that a mammogram contained a benign abnormality, which showed the same pattern of results as discussed below (see Appendix 6).

**Fig. 6:**
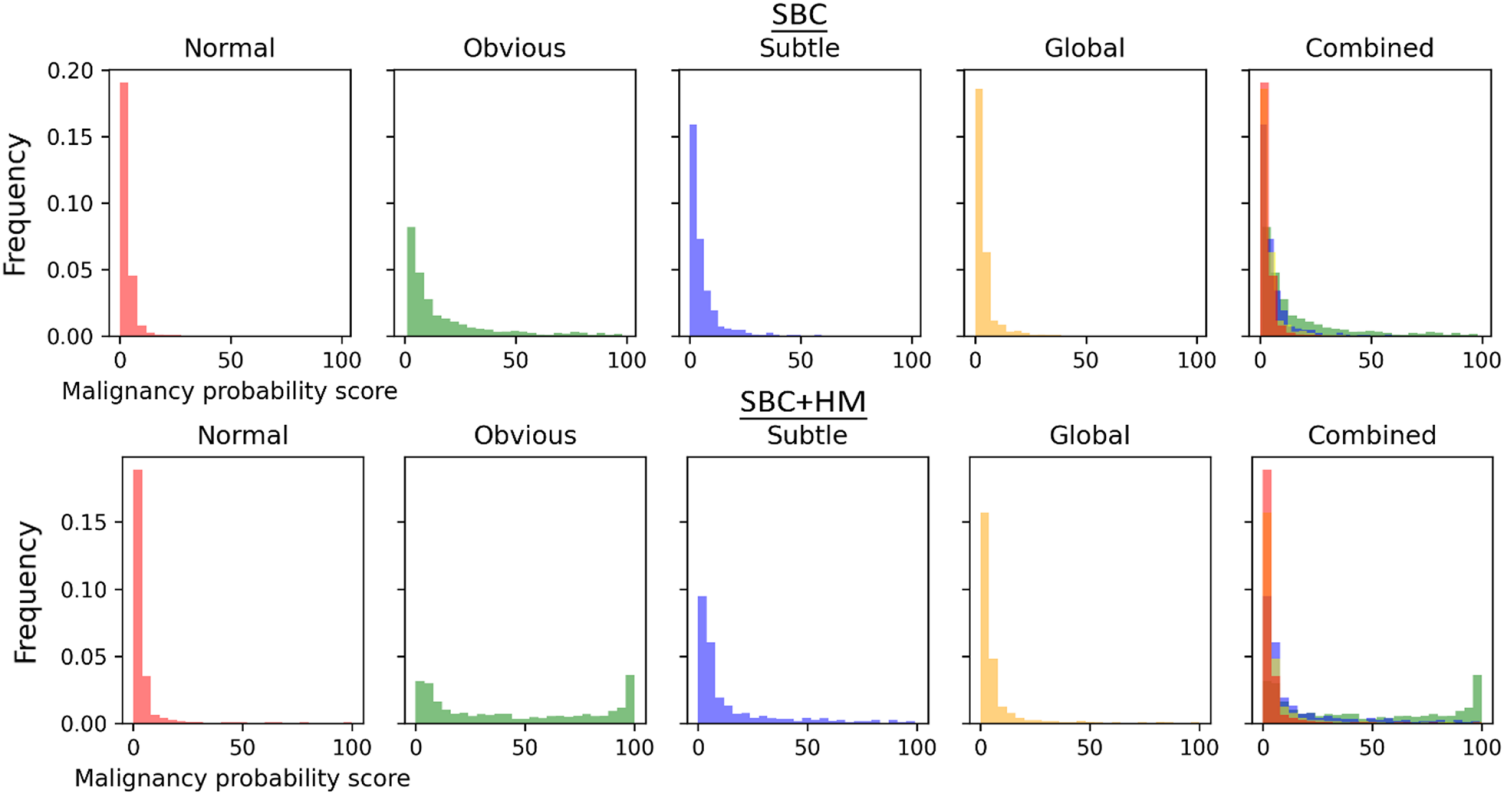
Distribution of single breast classifier (SBC) and SBC + Heatmap (SBC + HM) malignancy probability scores on the full image set of mammograms split into 25 bins for each of the image type categories, with a combined plot showing the overlap between normal (red), obvious (green), subtle (blue), and global (yellow) scores

Histograms of DNN malignancy probability scores show more overlap between the normal and global cases than between the obvious/subtle and normal cases (Fig. 6), indicating that both the SBC and SBC + HM were less able to distinguish global and normal from each other. The finding illustrates an apparent difficulty for the SBC and SBC + HM to distinguish the global gist signal of cancer compared to the visible obvious and subtle cancers.

Similarly, AUC calculations (Table 1) show that the SBC and SBC + HM both performed well in discriminating the obvious and subtle mammograms from the normal mammograms on malignancy probability, whereas AUC dropped considerably for the global mammograms, although it did remain above-chance levels for all except the malignancy-SBC on the global mammograms in the test set. The increase in AUC for SBC + HM shows that heatmaps improved the DNN’s ability to detect the probability of malignancy in mammograms, especially in more subtle cases. These results on our mammography image lend support to the reported increase in performance with the added heat map described in the original publication (Wu et al., 2019).

**Table 1:** AUCs for malignancy probability scores for the SBC and SBC + HM for obvious, subtle, and global mammograms versus the group of normal mammograms. This is calculated for the training set and the test set separately. Square brackets contain the lower and upper bands of 95% CIs

	Training set		Test set	
	SBC	SBC + HM	SBC	SBC + HM
Obvious	0.839 [0.842–0.854]	0.897 [0.885–0.909]	0.844 [0.772–0.916]	0.885 [0.824–0.946]
Subtle	0.689 [0.668–0.710]	0.738 [0.719–0.757]	0.701 [0.599–0.603]	0.803 [0.720–0.886]
Global	0.582 [0.563–0.601]	0.598 [0.579–0.617]	0.505 [0.408–0.602]	0.683 [0.596–0.770]

Most critically, the low or even at-chance performance (AUC: 0.505 SBC on the test set) on the globally abnormal mammograms shows that mammograms with the global signal of abnormality are especially obscure and difficult to detect. This adds to the significance of our finding that human observers were able to learn to detect abnormalities in these mammograms, performing above chance on the test set with which the SBC struggled severely. It also demonstrates that the chosen test set was representative of, or potentially even more difficult than, the overall mammography data set, and learning was not a result of coincidentally easier stimuli in the test set.

Next, a direct comparison of human and SBC scores was made to see if similar image statistics might be used by human observers and machine learning models. This was done by correlating the average rating from the ‘learner’ group of observers to the malignancy probability scores of the SBC and SBC + HM. Spearman’s rank correlations were performed between the DNN malignancy probabilities and the average of the human learner scores given pre- and post-perceptual training (Table 2). Before perceptual training, the correlation between SBC malignancy and human scores was non-significant (*p* = 0.137), while the correlation between SBC + HM and human scores was (*p* = 0.005). At the post-training test, the average human score across the 200 test mammograms correlated significantly with both the SBC and SBC + HM malignancy and benign scores (all *p* < 0.01, see Table 2). Comparing pre- and post-perceptual training correlations showed that the correlation coefficient increased after the human observers completed their perceptual training. After training, human scores more closely agreed with the classifier judgements—mammograms that were judged as more abnormal by humans also received higher malignancy probability scores.

**Table 2:** Spearman’s rank correlations between the average human learner score pre- and post-training of human observers, and the SBC/SBC + HM malignancy probability scores

		Pre-training		Post-training		Difference
		Correlation	*p* value	Correlation	*p* value	
SBC	Malignant	0.105	0.137	0.207	0.003	0.102
SBC + HM	Malignant	0.198	0.005	0.318	0.000	0.119

The finding that agreement between human and SBC scores increased after training has interesting implications. It suggests that the gist of abnormality signal learned by human observers during perceptual training is partially captured by the DNN as well. This adds validity to our findings, as the human observers learned signals that were also detected by an ‘expert’ in the form of a DNN, demonstrating they were able to learn image features of abnormality. Additionally, the finding that the correlation coefficient was markedly higher for the SBC + HM (0.318) than SBC (0.207) suggests that the added heatmap might capture additional perceptual features used by the trained human observers. This suggests that the SBC + HM and similar deep neural networks could be used to investigate the perceptual features in mammograms contributing to the gist signal, for example by performing network dissection, a technique where layers of the network are investigated to extract the content that is activating nodes in these layers (Bau et al., 2020).

## Conclusion

In conclusion, perceptual training with global feedback can result in the learning of the gist of a new category, although there are individual differences in both pre-training sensitivity to global structural regularities and ability to further learn the gist signal, and the new gist signal is poorly retained if exposure is not maintained. This suggests that gist categorization might be a case of ‘use it or lose it’, although retention or complete tuning of the visual system to a new category might be obtained after extended exposure. The exposure in our study only amounted to approximately 9 h task time, and 6470 instances viewed with feedback, which is substantially less than in real-world learning of gist categories.

Furthermore, human perceptual expertise on difficult, ambiguous cases containing only global signals of abnormality (contralateral, prior) is still not matched by state-of-the-art neural networks, as indicated by the markedly lower, or even at-chance performance of the DNN on mammograms with global abnormalities that human observers were able to learn in our perceptual training paradigm. The global signal of abnormality is extremely difficult to detect and requires considerable perceptual expertise. On the other hand, we also observed an increase in agreement between the human observers and DNN after perceptual training, which indicates a potential overlap in image statistics used to classify mammograms as normal or abnormal. Finding out what these image statistics are could teach us more about the gist of abnormality and could help find ways to improve image filtering for both human observers and machine learning models. Together, these findings solidly emphasize the need for continued research into medical perceptual expertise with human observers in its own right, especially into more ambiguous global signals that would be vital for early cancer detection. But it also reinforces the need of combining these lines of research with the thriving field of machine learning research, especially since recent research has suggested benefits of combining radiologists’ gist ratings with machine learning models to reach higher levels of performance than either could on their own (Gandomkar et al., 2021; Wurster et al., 2019).

We based our study on drawing a clear parallel between scene gist and the gist of abnormality in radiographs, and it would be beneficial to generalize the current results on learning to a wider area of gist extraction. The parallels between the two types of gist extraction would imply that the current findings of implicit learning should generalize to the learning of scene gist as well. However, as far as the authors are aware, this area has not yet been investigated in the known literature. A potential avenue to answering this question for scene gist could be developmental research with young children, especially as previous research has shown that infants already exhibit signs of statistical learning (Fiser & Aslin, 2002b). However, previous research on the development of rapid perceptual processing is very limited (but see Sweeny et al. (2015). Overall, developmental research often suffers from complications, such as communication of task instructions or difficulties in directing attention, a lack of control over previous exposure, individual differences, and other developmental processes occurring at the same time (Johnson, 2011; Maurer, 2013). These factors make it less suitable to investigate the acquisition of the gist of a novel category.

Overall, the current study shows a strong case for how implicit learning would allow the learning of a new category of any gist, including scenes. What is more, our finding that gist extraction abilities can develop separately from medical knowledge reinforces the viability of the idea, suggested by Voss et al. (2010), of using trained naïve observers, not to ‘usurp’ radiologists’ ratings, but to create a more accessible ‘model observer’ to use for further dissemination of the gist of abnormality signal. This training regime can be used for training of novice radiologists and screening radiographers or even as a refresher training for expert radiologists who over their careers see a considerable reduction in cases they read. Further research is needed to measure the effectiveness of our training paradigm on these populations, and to explore explanatory parameters for individual differences in pre-training performance, learning ability, and learning rate/speed, for example by investigating the potential variation in the length of perceptual training required to achieve perceptual learning across different participants.

## Figures and Tables

**Fig. 7:**
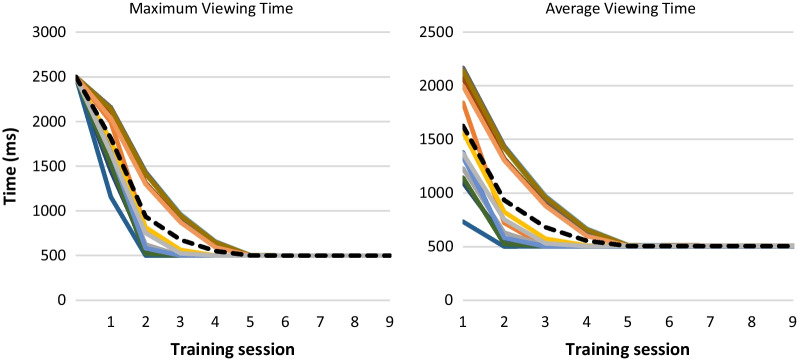
Maximum and average viewing time in milliseconds per participant at the end of each training phase. Maximum viewing time is calculated for the fourth block of the session. Individual lines are plotted, while the dashed black line represents the group average

**Fig. 8:**
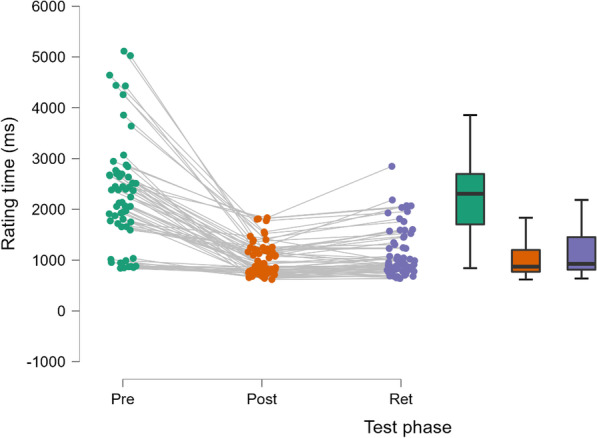
Individual average rating times are shown at pre-training (pre, green), post-training (post, orange), and retention (ret, purple) testing phases, as connected dot-clouds per participant-image type combination and boxplots to show both individual patterns and the population distributions

**Fig. 9:**
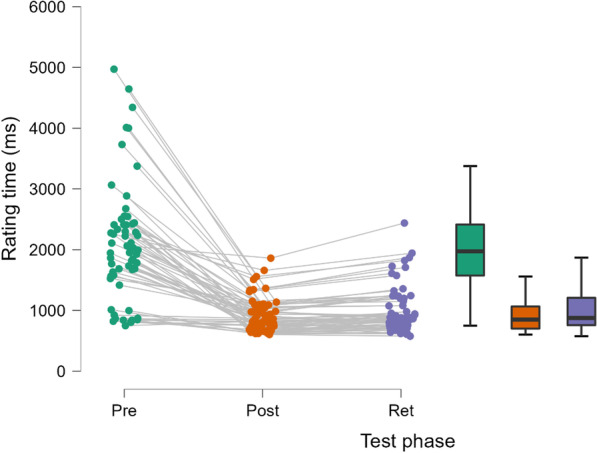
Individual median rating times are shown at pre-training (pre, green), post-training (post, orange), and retention (ret, purple) testing phases, as connected dot-clouds per participant-image type combination and boxplots to show both individual patterns and the population distributions

**Fig. 10:**
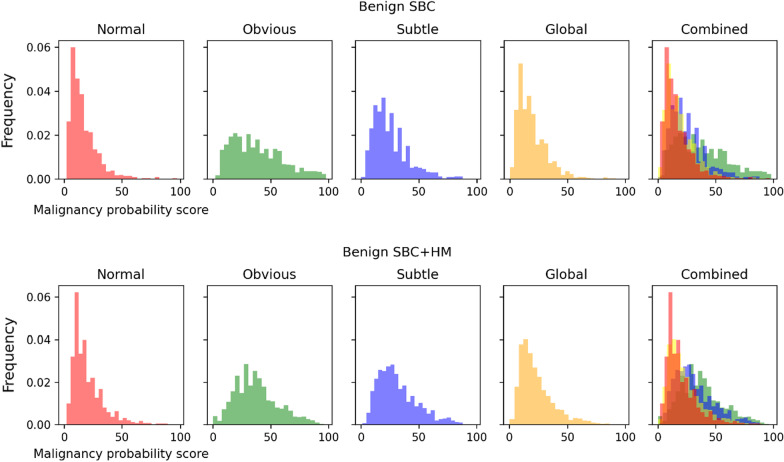
Distribution of single breast classifier (SBC) and SBC + Heatmap (SBC + HM) benign abnormality probability scores on the full image set of mammograms split into 25 bins for each of the image type categories, with a combined plot showing the overlap between normal (red), obvious (green), subtle (blue), and global (yellow) scores

## Data Availability

The raw data generated and analysed during the current study are available on our OSF repository, https://osf.io/mv47p/. These data are available under Creative Commons Attribution-NonCommercial-ShareAlike 2.0 UK: England & Wales (CC BY-NC-SA 2.0 UK).

## Appendix 1: Mammographic descriptors of obvious and subtle cases

Ductal carcinoma in situ (DCIS) grade can be classified as high, intermediate, or low. Percentages of DCIS grades in obvious and subtle mammograms can be found in Table 3). Tumour surfaces can be positive, negative, or borderline (not strongly + or −) for human epidermal growth factor receptor 2 (HER-2), and positive or negative for oestrogen and progesterone receptors (see Table 4 for percentages in the obvious and subtle cases). The presence or absence of these receptors in the tumour can impact both the cancer severity and viable treatment options. For example, the so-called triple-negative cancers, without HER-2, progesterone, and oestrogen receptors, currently lack approved targeted therapy and overall have poorer long-term outcomes (Sharma, 2016).

**Table 3:** Percentage of mammograms with a high, intermediate, low, or unassessed DCIS grade for the obvious and subtle subsets of the image set. Where descriptors were not available in the OPTIMAM database, the mammogram was classified as unassessed

	Obvious	Subtle
High	26.3	14.7
Intermediate	25.2	21.8
Low	8.7	7.9
Unassessed	39.7	55.6

**Table 4:** Percentage of mammograms that were positive, negative, borderline, or unassessed DCIS grade for the obvious and subtle subsets of the image set. Where descriptors were not available in the OPTIMAM database, the mammogram was classified as unassessed

	HER-2		Progesterone receptor		Oestrogen receptor	
	Obvious	Subtle	Obvious	Subtle	Obvious	Subtle
Positive	5.4	3.5	66.5	55.8	58.4	49.7
Negative	58.8	51.1	6.3	3.3	11.9	7.4
Borderline	0.5	0.1	N/A	N/A	N/A	N/A
Unknown	1.2	1.7	1.4	1.6	1.6	1.7

## Appendix 2: Engagement and attention in training phases

As mentioned in the main document, participants routinely used the spacebar to manually continue to the rating screen before reaching the maximum viewing time (2.5 s) in the first training phase. This occurred on average on 234 out of 720 trials (95% CI 138–330), indicating active engagement with the task instruction to view the mammogram until they formed a first impression to base their rating on. As a result, both average and maximum viewing time rapidly decreased, as is plotted in Fig. 7.

Additionally, participants viewed the feedback screen for an average of 741 ± 72.4 ms per trial across the 9 training phases, which is estimated to be sufficient to perceive the ‘right or wrong’ global feedback, due to the colour-coded and regular nature of the feedback text combined with the recency of the rating choice as feedback was shown immediately after confirming the rating. In conclusion, there was clear evidence of attention to and engagement with the training phases.

## Appendix 3: Log-linear likelihood ratios ROC curves

To evaluate whether individual participants’ performance was significantly above-chance the AUCs of log-linear likelihood (LLR) ROCs were compared to the AUC of the 95^th^ percentile AUC of simulated ROCs. As given in Table 5, the number of participants that performed above-chance increased from 5 to 11 overall after training, an increase driven by an increase from 1 to 7 out of 9 learners, while no change was observed for non-learners. This analysis shows that training caused most participants to outperform a very strict definition of chance levels, especially the subgroup of learners, in line with the significant testing phase effect observed for d’.

**Table 5:** Number of participants performing at above-chance levels (real AUC > 95^th^% simulated AUC) at each testing phase, split-up for learners, non-learners, and total

	Pre-training	Post-training	Retention
Learners	1 (11.11%)	7 (77.77%)	5 (55.55%)
Non-learners	4 (66.66%)	4 (66.66%)	2 (33.33%)
Total	5 (33.33%)	11 (73.33%)	7 (46.66%)

## Appendix 4: Effect of training on rating time

To evaluate if perceptual training affected participants’ decision-making speed, a 4 × 3 repeated measures ANOVA was conducted on the average rating time with the factors image type (normal, obvious, subtle, global) and testing phase (pre-training, post-training, and retention). Average rating time was significantly affected by test phase (F(1.08,15.10) = 25.590, *p* =  < 0.001 with Greenhouse–Geisser correction, η_p_^2^ = 0.646), but not by image type (F(3,42) = 1.631, *p* =  < 0.001, η_p_^2^ = 0.104), nor was there evidence for an interaction effect (F(6,84) = 0.594, *p* =  < 0.001, η_p_^2^ = 0.041). Rating time went down significantly after training compared to pre-training (difference = − 1291 ms, *p* < 0.001) and remained that way at retention (difference = − 1158 ms, *p* < 0.001), as shown by a simple contrast planned comparison. Due to the lack of evidence for an image type effect, the main effect of testing phase on average rating time is visualized in the bar graphs in Fig. 8. The same pattern persisted for median rating time.

Median rating time was also evaluated using a 4 × 3 repeated measures ANOVA with the factors image type (normal, obvious, subtle, global) and testing phase (pre-training, post-training, and retention). Median rating time was significantly affected by test phase (F(1.04,14.49) = 24.590, *p* =  < 0.001 with Greenhouse–Geisser correction, η_p_^2^ = 0.637), but not by image type (F(3,42) = 1.307, *p* = 0.285, η_p_^2^ = 0.085), nor was there evidence for an interaction effect (F(6,84) = 0.284, *p* = 0.943, η_p_^2^ = 0.020). Rating time went down significantly after training compared to pre-training (difference = − 1160 ms, *p* < 0.001) and remained that way at retention (difference = − 1069 ms, *p* < 0.001), as shown by a simple contrast planned comparison and visualized in Fig. 9. Thus, participants took significantly less time to make rating decisions after completing their training.

## Appendix 5: Criterion for learners and non-learners

For learners, it showed that the criterion was affected by both image type (F(2, 16) = 13.169, *p* < 0.001, ηp2 = 0.622) and testing phase (F(2,16) = 12.509, *p* < 0.001, ηp2 = 0.610), without interaction effect (F(4,32) = 0.223, *p* = 0.924, ηp2 = 0.027). Planned comparisons with a simple contrast showed that post-training criterion was significantly lower (more liberal) than pre-training levels (estimate: − 0.345, t(16) = 4.703, *p* < 0.001), and remained this way at retention (estimate: − 0.280, t(16) = 3.826, *p* = 0.001). However, for non-learners, criterion was not affected by image type (*F*(1.091, 5.455) = 3.409, *p* = 0.118, ηp2 = 0.405) nor testing phase (*F*(2,10) = 2.002, *p* = 0.186, ηp2 = 0.286), but did show an interaction effect (*F*(4,20) = 4.254, *p* = 0.012, ηp2 = 0.460).

## Appendix 6: DNN probability of benign abnormality

Histograms of DNN benign probability scores show more overlap between the normal and global cases than between the obvious/subtle and normal cases (Fig. 10), indicating that both the SBC and SBC + HM were less able to distinguish global and normal from each other. Similar to the malignancy probability scores, this again illustrates the difficulty for the SBC and SBC + HM to distinguish the global gist signal of cancer compared to the visible obvious and subtle cancers.

AUC calculations for benign probabilities (Table 6) show that the SBC and SBC + HM both performed well in discriminating the obvious and subtle mammograms from the normal mammograms on malignancy probability, whereas AUC dropped considerably for the global mammograms, although it did remain above-chance levels (~ 0.55).

**Table 6:** AUCs for the probability of benign abnormality for the SBC and SBC + HM for obvious, subtle, and global mammograms versus the group of normal mammograms. This is calculated for the training set and the test set

	Training set		Test set	
	SBC	SBC + HM	SBC	SBC + HM
Obvious	0.817 [0.801–0.833]	0.818 [0.802–0.834]	0.818 [0.739–0.897]	0.785 [0.698–0.872]
Subtle	0.701 [0.681–0.721]	0.670 [0.649–0.691]	0.670 [0.563–0.777]	0.764 [0.673–0.855]
Global	0.569 [0.550–0.588]	0.555 [0.536–0.574]	0.555 [0.459–0.651]	0.547 [0.451–0.643]

Spearman’s rank correlations between the DNN malignancy probabilities and the average of the human learner scores given pre- and post-perceptual training (Table 7) showed a marked increase in correlation after perceptual training. After training, human scores more closely agreed with the classifier judgements—mammograms that were judged as more abnormal by humans also received higher benign abnormality probability scores.

**Table 7:** Spearman’s rank correlations between the average human learner score pre- and post-training, and the SBC/SBC + HM probabilities of benign abnormality

		Pre-training		Post-training		Difference
		Correlation	*p* value	Correlation	*p* value	
SBC	Benign	0.286	0.000	0.373	0.000	0.087
SBC + HM	Benign	0.280	0.000	0.402	0.000	0.122

## Appendix 7: DNN correlation with non-learners

Correlating SBC scores with the average ratings of the learner group showed that the correlation went up post-training. The same correlations were performed for the average ratings of the non-learners (Table 8).

**Table 8:** Spearman’s rank correlations between the average human non-learner score pre- and post-training, and the SBC/SBC + HM probabilities of malignant or benign abnormality

		Pre-training		Post-training		Difference
		Correlation	*p* value	Correlation	*p* value	
SBC	Malignant	0.158	0.026	− 0.038	0.592	− 0.196
SBC + HM	Malignant	0.301	0.000	0.099	0.161	− 0.201
SBC	Benign	0.310	0.000	0.104	0.142	− 0.206
SBC + HM	Benign	0.342	0.000	0.105	0.140	− 0.237

These results show two things. Firstly, before training, the correlation between SBC + HM malignancy predictions and the non-learners was 0.301, compared to 0.198 for learners. This suggests that the non-learners might have started out sensitive to part of the same signals used by the SBC, and especially the SBC + HM. Potentially, this could be caused by more focus on localized signals, as implied by the increased correlation with the added heatmap—which adds scrutiny to local features. Secondly, the correlation between non-learner and SBC goes down after training and becomes non-significant for all four comparisons. This was unexpected and could be the result of a maladaptive learning strategy, where non-learners incorrectly establish certain perceptual features as normal/abnormal and this leads them to not only fail at learning, but additionally diverge from the SBC predictions. However, since this data set only contained six non-learners, a larger, more structured approach would be needed to further investigate potential maladaptive strategies in such a perceptual learning task.

## Abbreviations

AUC: Area under the curve
ROC: Receiver operating characteristic
SBC: Single breast classifier
SBC + HM: Single breast classifier plus heatmap
